# UV Absorption Spectroscopy in Water-Filled Antiresonant Hollow Core Fibers for Pharmaceutical Detection

**DOI:** 10.3390/s18020478

**Published:** 2018-02-06

**Authors:** Mona Nissen, Brenda Doherty, Jonas Hamperl, Jens Kobelke, Karina Weber, Thomas Henkel, Markus A. Schmidt

**Affiliations:** 1 Leibniz Institute of Photonic Technology, Albert-Einstein-Str. 9, 07745 Jena, Germany; mona.nissen@leibniz-ipht.de (M.N.); brenda.doherty@leibniz-ipht.de (B.D.); Jonas.Hamperl@leibniz-ipht.de (J.H.); jens.kobelke@leibniz-ipht.de (J.K.); karina.weber@leibniz-ipht.de (K.W.); thomas.henkel@leibniz-ipht.de (T.H.); 2 Otto Schott Institute of Materials Research (OSIM), Friedrich Schiller University of Jena, Fraunhoferstr. 6, 07743 Jena, Germany

**Keywords:** optical fiber sensor, water monitoring, microstructured optical fibers, fiber-based optofluidics

## Abstract

Due to a worldwide increased use of pharmaceuticals and, in particular, antibiotics, a growing number of these substance residues now contaminate natural water resources and drinking supplies. This triggers a considerable demand for low-cost, high-sensitivity methods for monitoring water quality. Since many biological substances exhibit strong and characteristic absorption features at wavelengths shorter than 300 nm, UV spectroscopy presents a suitable approach for the quantitative identification of such water-contaminating species. However, current UV spectroscopic devices often show limited light-matter interaction lengths, demand sophisticated and bulky experimental infrastructure which is not compatible with microfluidics, and leave large fractions of the sample analyte unused. Here, we introduce the concept of UV spectroscopy in liquid-filled anti-resonant hollow core fibers, with large core diameters and lengths of approximately 1 m, as a means to overcome such limitations. This extended light-matter interaction length principally improves the concentration detection limit by two orders of magnitude while using almost the entire sample volume—that is three orders of magnitude smaller compared to cuvette based approaches. By integrating the fibers into an optofluidic chip environment and operating within the lowest experimentally feasible transmission band, concentrations of the application-relevant pharmaceutical substances, sulfamethoxazole (SMX) and sodium salicylate (SS), were detectable down to 0.1 µM (26 ppb) and 0.4 µM (64 ppb), respectively, with the potential to reach significantly lower detection limits for further device integration.

## 1. Introduction

The contamination of drinking water and water supplies by otherwise widely accepted substances, such as pharmaceuticals, forms one severe threat of our modern society. The presence of such substances presents unforeseen consequences for human health, such as direct intoxication or, even more severe, the formation of multi-resistant pathogens [1,2,3,4], potentially rendering currently used antibiotic treatments ineffective. These highly relevant issues define a clear need to develop low cost, straightforward-to-apply, but also highly sensitive, methods for water monitoring [5]. Besides schemes such as [6,7], ultraviolet (UV) spectroscopy has turned out to be a highly sensitive and quantitative analysis method, due to the characteristic fingerprint absorption features of all organic substances resulting from electronic resonances at wavelengths <300 nm [8,9]. For example, the widely used pain reliever, Paracetamol, exhibits a characteristic absorption feature at 243.57 nm [10].

Generally, the linear absorption of light inside a medium, that is transparent over the device length considered and includes species which strongly absorb, is given by the well-known Beer–Lambert law:

(1) $$\frac{P\;}{\;P_{0}}=\;10^{-\varepsilon \left(\lambda \right)cL}$$

where *P* and *P*_0_ are transmitted powers through contaminated and pure water, respectively, *ε*(*λ*) is the wavelength dependent molar absorptivity of the analyte (in L/(mol·cm)), *c* is the concentration of the absorbing substance (in mol/L (=M)) and *L* is the light-matter interaction length (in cm). It is evident from Equation (1) that increasing the length effectively allows detection of lower concentration levels of an analyte as, e.g., shown in [11]. By implementing the fiber-based approach discussed in this work, light-matter interaction lengths are in the order of 1 m, which is about 100 times longer compared to typical cuvette-based systems (exhibiting path lengths of ~1 cm). This allows, in principle, the detection of 100 times smaller analyte concentrations, while requiring 1000 times lower analyte volume.

Due to the low refractive index (RI) of water (~1.33 at visible wavelengths), guiding light over long distances inside a water-core waveguide remains a key challenge within optofluidics, since very few cladding materials promote total internal reflection as a guidance principle. An example is Teflon AF [11]) which exhibits high optical attenuation due to surface roughness and thus demands mm core diameters. One solution to this problem is hollow-core optical fibers, which allow light guidance inside a low index core by using, e.g., the photonic band gap effect [12], omnidirectional reflection [13,14], effective medium reflection [15,16], or low density of states [17]. Recently, anti-resonant hollow core fibers (ARHCFs) have garnered attention [18] due to their simplified fabrication scheme and low optical loss across large spectral windows [19], including the UV spectral domain [20,21]. Due to the large diameter of the central core section (typically in the order of a few tens of micrometers), filling and exchanging liquids inside the core is straightforward and requires mere seconds to minutes [22]. For instance, the time to exchange the entire liquid volume (viscosity η) inside a capillary (length L, radius R) is given by $T_{e}=8\eta L^{2}/\left(R^{2}\Delta p\right)$ with the pressure difference, Δp [23]. Assuming experimentally relevant numbers ($\eta =10^{-3}\;\mathrm{Pa}\cdot s$ (water), L=1 m, R=20 µm and Δp=10 bar), the exchange time is only $T_{e}=20\;s$.

Another feature of the ARHCF concept is that the typical volume of a liquid used in cuvette-based photometric experiment (~5 cm^3^) is reduced by a factor of 1000 when using ARHCFs (~0.007 cm^3^), demanding a volume that is 100 times smaller than that of typically used optofluidic waveguides (e.g., ~0.7 cm^3^ for the Teflon AF waveguide (inner diameter 1 mm) used in [11]). Moreover, it is important to note that due to the large concentration of the electromagnetic energy in the core section (i.e., more than 99.99% of the modal field overlaps with the medium inside the core) itis possible to directly employ Equation (1).

## 2. Materials and Methods

### 2.1. Antiresonant Hollow Core Fiber

**Figure 1:**
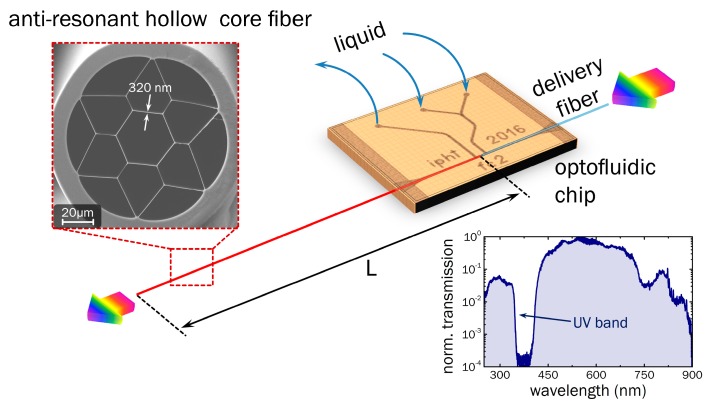
Schematic of the concept of UV spectroscopy using water-filled anti-resonant hollow core fibers (**red**), including the microfluidic chip (**orange**) and the delivery fiber (**light blue**). The two insets show a scanning electron micrograph image of the cross section of the silica microstructured fiber (**upper left**, the number refers to the average strand thickness in nm) and an example sample transmission spectrum (normalized to the maximum transmission value at 560 nm) where the fiber is filled with water (**lower right**).

The waveguide forming the basis of the presented detection concept (Figure 1) is a silica ARHCF with effective single-mode guidance and a low-loss transmission window in the UV (a cross section of which is shown in the scanning electron micrograph (SEM) in Figure 1) [20]. The fiber contains a hexagonally shaped inner core with a diameter of approximately 30 μm, surrounded by essentially a dielectric ring with a thickness of *t* = (320 ± 20) nm (the latter number results from thickness variations occurring during the fiber fabrication that is reported elsewhere [20,21]). The outer diameter of the fiber amounts to 125 μm, while the solid outer silica cladding has a thickness of 10 μm. The operation principle of the ARHCF can be understood on the basis of interface reflection: assuming a plane wave impinging on a thin silica film of thickness identical to that of the core-ring, close-to-unity reflection can be obtained due to the interference between the two waves reflected at both interfaces, i.e., the initial wave is anti-resonant with the mode inside the film. It was shown for a tube-type single ring ARHCF that modal attenuation scales with *R*^−4^ [24]. The wavelengths at which the core mode is resonant with the mode inside the film—i.e., the electromagnetic energy can transversely dissipate—are given by

(2) $$\lambda_{m}=\frac{2t\;}{m}\sqrt{n_{s}^{2}-n_{h}^{2}}$$

with the resonance order *m*, and the RIs of silica and the medium inside the holes, *n_s_* and *n_h_*, respectively (the derivation of this equation can be found in the Supplementary Materials and in ref. [25]). The lower right inset of Figure 1 shows the raw transmission spectrum of the studied ARHCF in the water-filled case (incl. light source and delivery/collection fibers), i.e., for *n_h_* = 1.33 [26], revealing a transparency window below 340 nm—suitable for the absorption spectroscopy of pharmaceutical substances.

### 2.2. Optofluidic Chip

From the application perspective, a key question is how to fill and exchange the analyte solution inside the ARHCF while simultaneously coupling light in and out of the fiber. Here, our experience has shown that fiber-to-fiber butt coupling is the most efficient and reliable way to fulfill both conditions. The input section of the fiber was therefore integrated into an optofluidic chip design that is compatible with common microfluidics. This protects the coupling junction against external influences and facilitates straightforward liquid filling and exchange, while at the same time enabling monitoring of the light transmission at UV wavelengths.

**Figure 2:**
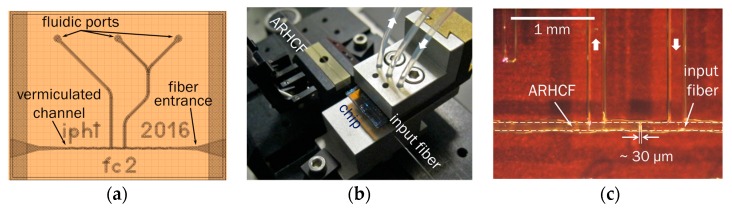
(**a**) Design sketch of the optofluidic chip, which allows light coupling into the anti-resonant hollow core fibers (ARHCF) via a delivery fiber, while providing simultaneous access to fluidics through the fluidic ports (one square scales to 0.5 mm); (**b**) Image of the optofluidic chip with delivery fiber, ARHCF, and tubing connected; (**c**) Enlargement of the chip section around the butt coupling location. The dashed lines indicate the outer boundaries of both fibers. In (**b**,**c**), the thick white arrows indicate the direction of liquid flow.

The optofluidic chip was fabricated from two 0.7 mm thin glass plates with etched channels of roughly 64 μm depth, which were glued together, with the channel openings facing each other. This yields a total channel diameter of approximately 128 μm inside the chip, which is suitable to support fibers of diameters of 125 µm. The vermiculated structure of the channel (design sketch in Figure 2a) was introduced to keep the fibers aligned in the transverse direction, while still enabling fine tuning of position along the channel. The etched channels are optofluidically accessed by ultrasonically drilling fluidic ports into the glass, using an Ultrasonic Disc Cutter (Gatan), equipped with a 0.5 mm drilling tool, so that they can be connected with tubes to specific chip holders (Figure 2b).

A monolithic and integrated design was achieved by fixing the ARCHF together with a light delivery fiber in series inside the chip channel (Figure 2c). The key idea that enables liquid access to the hollow sections of the ARHCF while maintaining high light in coupling efficiencies relates back to the fact that the numerical aperture of the modes inside the ARHCF are comparably small (<0.1), allowing micrometer distances between delivery and ARHCF. It is important to note that the anti-resonance effect allows light guidance in the central core in cases where all holes are filled with analyte. Thus, no sophisticated post-processing is required to contain the liquid within the core section only. Preliminary experiments have shown that fiber gap separations in the order of a few tens of micrometers are feasible when butt coupling from an in-house fabricated UV guiding multimode fiber (core and outer diameters: 15 μm core and 125 μm; numerical aperture 0.1; length 50 cm; loss spectrum of this fiber can be found in the Supplementary Materials). The overall coupling efficiency of the whole system (delivery fiber and ARHCF) was approximately 23%. The preparation of the monolithic design included the following steps: The two fibers were carefully cleaned and cleaved, so that no contaminants or dust were introduced into the chip and the fiber end faces were in optimal condition. Then, with the help of high precision positioning stages, the fibers were threaded into the channel from opposite sides (Figure 2c), with the ARHCF coming from the left (contrary to the liquid flow direction) and the input fiber from the right. Both fiber end faces were moved to a coupling position between the inflow and outflow channels. At the same time, the free end of the delivery fiber was connected to a light source and the output end face of the ARHCF was imaged by an objective onto a camera CCD chip (Thorlabs CMOS USB 2.0 camera, model “DCC1240C”). In this way, the coupling was optimized while observing the mode transmitted through the ARHCF. Finally, the fibers were mechanically fixed by UV curing glue at their chip exit points. Final end face separations numbered a few tens of micrometers (Figure 2c), yielding good modal images and high throughput while maintaining sufficient space for the analyte solution to enter the holey sections of the ARHCF.

### 2.3. Spectroscopic Setup

**Figure 3:**
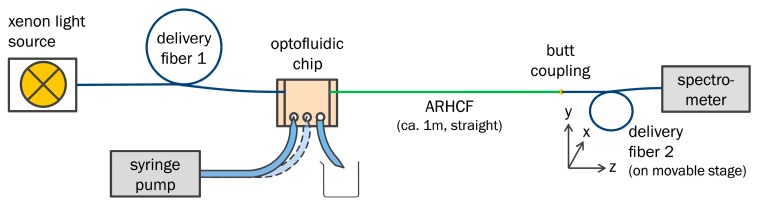
Schematic setup used for recording spectra. White light is delivered to the chip by a broadband xenon source through a UV guiding multimode fiber (15 µm core diameter, NA 0.1); liquids are introduced via syringe pump and tubing. From the ARHCF end face, the light is butt coupled to a second length of the same multimode fiber, delivering it to the spectrometer (Instrument Systems “Spectro 320”).

As with any spectroscopic device, the setup used for absorption spectroscopy measurements consisted of a light source, the sample mounted into the aforementioned chip environment, and a detector (schematic in Figure 3). For illumination, a high-power xenon light source (Mikropack HPX-2000, Ocean Optics, Ostfildern, Germany) was applied, which has a spectral emission range from 185 to 2000 nm, according to the manufacturer. Below 250 nm, however, the emitted power was insufficient for conducting spectroscopic measurements. The light emitted by the source was coupled to the analyte-filled ARHCF via the delivery fiber and again, butt coupled to a collecting fiber, identical to the delivery fiber, at the output of the ARHCF, in order to guide the light to a spectrometer (Spectro 320, Instrument Systems, Munich, Germany, Figure 2c). Only the input side of the ARHCF was integrated into the optofluidic chip environment, since the number of available chips was limited at the time of the experiment and any air bubbles could be straightforwardly removed from the sections filled fiber holes via pumping. The filling of the liquid into the 1 m long fiber was achieved by a syringe pump that was connected via tubing and holder to the optofluidic chip. Before any spectral measurement, the output intensity was maximized by fine-adjusting the butt coupling between ARHCF and collection fiber, while observing the transmitted intensity at a certain wavelength (usually 330 nm which was within the ARHCF’s UV transmission window). Together with a visual microscopic inspection of the filling process from the side, this procedure ensures bubble-free and complete filling/exchange of the liquid in the holey fiber sections, to reach the best possible performance in terms of transmittance.

**Figure 4:**
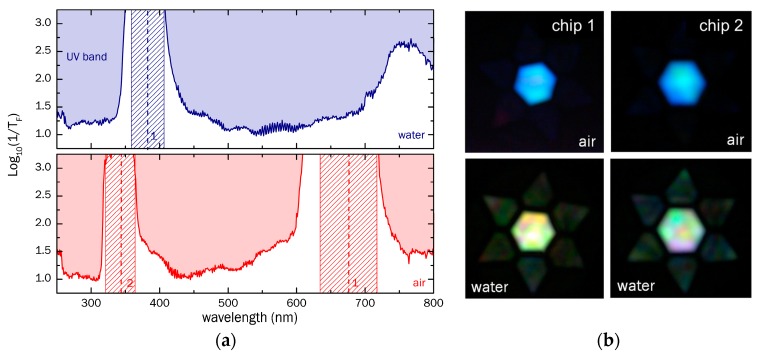
(**a**) Spectral distribution of the logarithmic inverse fiber transmittance (logarithmic scale) of the water (**top**, blue) and air (**bottom**, red) filled ARHCF, corresponding to the qualitative loss distributions. The dashed lines indicate the resonant wavelengths predicted by Equation (2) for given mode orders, whereas the hachured regions correspond to the resonance uncertainties imposed by strand thickness variations of ±20 nm; (**b**) Modal near field images of the modes supported by the ARHCF of two individual fiber-chip ensembles.

Here, the fiber transmittance *T_F_* (i.e., transmission solely originating from the modal properties of the ARHCF) is obtained by dividing the respective raw power spectrum $P_{raw}\left(\lambda \right)$ by that of the light source spectrum, $P_{LS}\left(\lambda \right)$ (spectrum shown in the Supplementary Materials in Figure S1a) and by the transmittance of a 50 cm long delivery fiber $T_{DF}\left(\lambda \right)=10^{-\alpha_{DF}\cdot 50\;\mathrm{cm}}$ (loss spectrum shown in the Supplementary Materials in Figure S1b) leading to $T_{F}=P_{raw}/P_{DF}T_{DF}$. The logarithmic inverse fiber transmittance $t_{F}=1/T_{F}$ of both water and air-filled configurations are shown in Figure 4a (top/blue: water, bottom/red: air). In this inverse representation, the locations of low-loss transmission bands (low inverse transmittance values) and high-loss regions (high inverse transmittance values) resulting from the strand resonances are easily visible. The resonant wavelengths predicted by Equation (2) are marked by dashed vertical lines in Figure 4a along with uncertainty regions (dashed areas) due to fabrication induced variations in the strand thicknesses (*t* = (320 ± 20) nm). The experimental results correspond well to the theory and illustrate that the visible transmission window of the air-filled case (380–600 nm) is blue-shifted to the UV region in the situation of the ARHCF being filled with water (low inverse transmittance for *λ* < 320 nm), which overall, is a result of the dependence of the strand resonance on core RI ($\lambda_{m}^{w}/\lambda_{m}^{a}=\sqrt{\left(n_{s}^{2}-n_{w}^{2}\right)/\left(n_{s}^{2}-1\right)}\approx 0.55$). For wavelengths >400 nm, no further resonance is found for the water-filled ARHCF, with the overall inverse transmittance increasing towards longer wavelengths. This effect is explained by the diminishing impact of the interference between the waves reflected at the two interfaces of the inner ring, since the phase difference between the two waves vanishes for large ratios of wavelength and film thickness. It is important to note that the overall magnitude of the inverse fiber transmittance within the transmission bands is roughly the same for both situations (inverse transmittance $\log_{10}\left(t_{F}\right)\;\approx 1.1–1.2$), revealing that the introduction of water into the ARHCF does not induce additional losses. The slight increase in the inverse transmittance at UV wavelengths in the case of the water filled fiber is presumably associated with the reduced RI contrast between core material and dielectric strands. An additional contribution of water to the modal attenuation can be excluded, since the extinction of water for wavelengths >200 nm is <10^−3^ cm^−1^ up to the infrared wavelength regime [27]. A qualitative change in the spectrum is also shown by the mode images in Figure 4b, taken by replacing the collection fiber and spectrometer by an objective and a camera (setup in Figure 3).

### 2.4. Sample Substances

Here we have chosen the antibiotic, sulfamethoxazole (SMX) and the analgesic and antipyretic, sodium salicylate (SS), as test substances to evaluate the concept of UV spectroscopy in ARHCF. Both species show absorption features within the transmission bands of the fiber used. Traces of SMX have already been detected in environmental water at many locations in Europe, America and Asia [2,3,28,29], whereas SS was chosen due to its pronounced absorption peak at a very central position of the UV transmission window of the water-filled ARHCF. The molar absorptivity, *ε*, at the relevant peak position amounts to 3600 L/(mol·cm) for SS (at 296 nm) [30] and approximately 16,700 L/(mol·cm) for SMX (at 265 nm).

**Figure 5:**
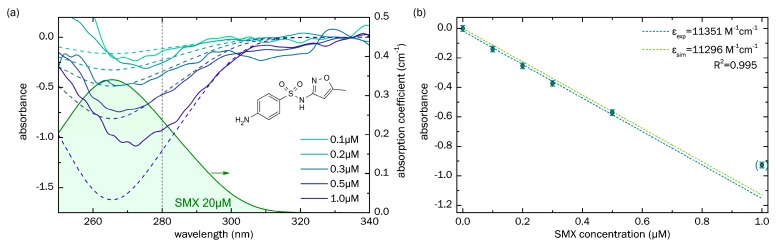
Absorption properties of sulfamethoxazole (SMX) inside the optofluidically integrated ARHCF. (**a**) Spectral distribution of the absorbance for a series of different SMX concentrations (indicated by the different colors), measured using the water-filled ARHCF. Solid lines represent experimental results, dashed lines represent the values of simulations. The green filled curve shows an example absorption spectrum of SMX, measured using a solution concentration of 20 μW and bulk UV spectroscopy. The chemical composition is shown on the upper right. The transmission band of the water-filled ARHCF spans across the entire presented spectral interval. (**b**) Absorbance, as a function of SMX concentration (calibration curve), at a wavelength of 280 nm (indicated in (**a**) by the vertical gray dashed line). The dark cyan points refer to the experimental data, including a relative transmittance error of 10%, with the dark cyan dashed line representing a linear fit (correlation coefficient $R^{2}=0.995$), allowing the determination of the measured (decadic) molar absorptivity ($\varepsilon_{A,exp}=11,351\;M^{-1}{\mathrm{cm}}^{-1}$ ). Due to an insufficient dynamic range, the measurement point at c=1 µM (point in brackets) was excluded from the fitting procedure. The light green dashed curve refers to the corresponding simulations with a (decadic) molar absorptivity of $\varepsilon_{A,sim}=11,296\;M^{-1}{\mathrm{cm}}^{-1}$.

**Figure 6:**
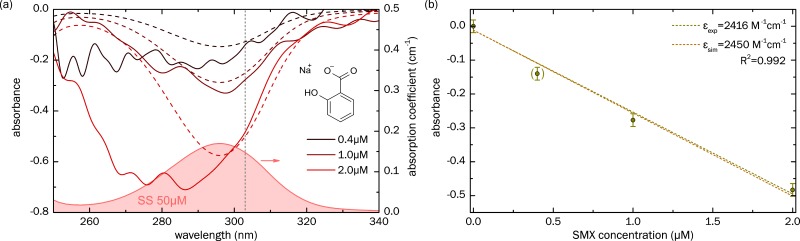
Absorption properties of sodium salicylate (SS), measured using the integrated ARHCF concept. (**a**) Spectral distribution of the absorbance for various SS concentrations (indicated by the different colors), measured by using the water-filled ARHCF (solid lines: experiments, dashed lines: simulations). The red filled curve shows an example absorption spectrum of SS at a concentration of 50 μW. The chemical composition is shown on the right; (**b**) Absorbance, as a function of SS concentration (calibration curve), at a wavelength of 303 nm (indicated in (**a**) by the vertical gray dashed line). The dark yellow points refer to the experimental data (relative transmittance error 10%) and the corresponding dashed line refers to a linear fit of the data points (correlation coefficient $R^{2}=0.992$), allowing the determination of the measured (decadic) molar absorptivity ($\varepsilon_{A,exp}=2416\;M^{-1}{\mathrm{cm}}^{-1}$ ). Due to fluctuations, the measurement point at c=0.4 µM (point in brackets) was excluded from the fitting procedure. The brown curve shows corresponding simulations, including a (decadic) molar absorptivity of $\varepsilon_{A,sim}=2450\;M^{-1}{\mathrm{cm}}^{-1}$.

Both sample substances were spectroscopically investigated in the form of water-based solutions. SMX was available in powder form and was directly dissolved using ultrasonic treatment. From a highly concentrated solution, all other solutions were prepared in a dilution series and subsequently checked in a double-beam UV-VIS spectrophotometer (Jasco V660) using a cuvette of 1 cm length, with pure water as the reference (example absorption spectrum shown in Figure 5a). For the lowest solution concentrations, the absorption peak was within the noise level of the spectrometer and could not be determined anymore. Sodium salicylate (SS), or salicylic acid sodium salt, was not available in powder form, but was obtained by mixing equal molar concentrations of salicylic acid, C_6_H_4_(OH)COOH, and sodium hydroxide, NaOH, in water solutions. The product was a solution of SS with half the molar concentration of the reagents. Similar to SMX it was diluted to different levels, and the concentrations of the prepared solutions were again verified by spectrophotometry, as long as the measured peak absorption was above the noise limit (example is shown in Figure 6a).

### 2.5. Measurement Procedure and Data Processing

For each individual spectroscopic measurement of a water-dissolved substance at a certain concentration, the ARHCF was first rinsed with pure water, and a reference spectrum was taken. Subsequently, the sample solution containing the analyte was pumped into the fiber and the transmission spectrum was measured. The spectroscopic raw data was smoothed and normalized. In the case of SMX, the spectra were normalized to the maximum value inside the UV transmission window of the ARHCF, around 335 nm, since the substance absorption is negligibly small at that wavelength (Figure 5a). For SS the local intensity maximum in the visible spectral domain closest to 468 nm was taken as the reference wavelength. From the processed data, the final wavelength dependent absorbance, A, was calculated in accordance with Equation (1), by taking the decadic logarithm of the power ratio between the power spectra of water and analyte solution, which is correlated to the (decadic) molar absorptivity, as follows (according to the International Union of Pure and Applied Chemistry (IUPAC) glossary of terms used in photochemistry [31]:

(3) $$A={\mathrm{Log}}_{10}(P_{\mathrm{water}}/P_{\mathrm{solution}})=\varepsilon_{A}cL$$

A preliminary data analysis revealed that output power varies by about 10% from measurement to measurement, defining the overall measurement uncertainty. We believe that by further integrating the ARHCF—i.e., by using a second optofluidic chip at the output side of the fiber—the fluctuations and thus, the measurement uncertainty, will strongly reduce. The limit-of-detection (LoD) has been calculated in accordance with the definition by the International Union of Pure and Applied Chemistry (IUPAC), which includes three times the standard deviations of the blank measurements $s_{A}^{blank}$ and the slope of the respective calibration curve $\beta =\varepsilon_{A}L$, as follows: $LoD=3s_{A}^{blank}/\beta$.

## 3. Results

In the following, the results of the in-fiber transmission measurements of the two investigated pharmaceutical substances are presented and compared to corresponding calculated spectra.

### 3.1. Sensing of Sulfamethoxazole

The solid lines in Figure 5a show the spectral distributions of the absorbance measurements for a series of SMX concentrations, from 0.1 μM to 1.0 μM, in pure water, using the optofluidically integrated ARHCF concept. The overlap with corresponding simulated curves is particularly good in the region between 275 nm and 300 nm, where the light source output power was sufficiently high. Above 300 nm, some measured curves show relatively strong deviations from simulations, which can most likely be explained by measurement-to-measurement variation due to slightly different outcoupling conditions from the ARHCF and the overall low absorption of the SMX at such long wavelengths, making the determination of the transmission more susceptible to spectrometer noise.

As the next step in the analysis, we created a calibration diagram (Figure 5b), which required plotting the absorbance against the SMX concentration at a selected wavelength (here we chose 280 nm, indicated by the vertical gray line in Figure 5a). The results clearly suggest a linear dependence, in accordance with Equation (3), allowing us to linearly fit the data points with high accuracy ($R^{2}=0.992$) to obtain the (decadic) molar absorbtivity. Due to an insufficient dynamic range, the point at c=1 µM (point in brackets) was excluded from the fitting procedure. The resulting value for the experimentally determined absorbtivity ($\varepsilon_{A,exp}=11,351{\;M}^{-1}{\mathrm{cm}}^{-1}$) agrees well with that obtained from simulations ($\varepsilon_{A,sim}=11,296{\;M}^{-1}{\mathrm{cm}}^{-1}$) and clearly confirms that the concept of UV spectroscopy using ARHCF is a reasonable approach for detecting small traces of substances, quantitatively. Using the calibration curve and the standard deviation of blank measurements, the LoD for SMX is calculated as $LoD_{SMX}=0.05\;µM$.

### 3.2. Sensing of Sodium Salicylate

Similar to the SMX measurements, the spectral distributions of the absorbance of a series of sodium salicylate (SS) solutions at different concentration levels were measured using the solution-filled ARHCF, and the results were compared to simulations (Figure 6a). In particular, the two highest concentrations clearly reveal the impact of SS on the absorbance and agree well with simulations between 295 nm and 310 nm. The curve corresponding to c=0.4 µM, however, shows rather strong fluctuations, presumably since the absorption-induced change in the transmitted power is too small and thus, this data point was omitted in the discussion of the calibration plot. It is important to note that the peak of the absorption coefficient of SS (red curve in Figure 6a) is located at around 296 nm, which is much longer than SMX (265 nm, green curve in Figure 5a), and thus, we decided to conduct the calibration analysis at 303 nm. Similar to SMX, a linear dependence of absorbance vs. SS concentration is observed (dark yellow points in Figure 6b), with the linear fitting procedure (indicated by the dashed dark yellow line in Figure 6b, $R^{2}=0.992$) yielding a molar absorbtivity of $\varepsilon_{A,exp}=2416{\;M}^{-1}{\mathrm{cm}}^{-1}$, which agrees well with the simulated value ($\varepsilon_{A,sim}=2450{\;M}^{-1}{\mathrm{cm}}^{-1}$, brown line in Figure 6b), again showing that the concept of UV-spectroscopy using integrated ARHCF is reasonable. The LoD of SS accounts to $LoD_{SS}=0.23\;µM$, which is about 4.6 higher than SMX, due to the different slope of the calibration curve.

## 4. Discussion and Conclusions

The presented results clearly show that the concept of UV spectroscopy in ARHCF is suitable for the detection of water contaminants at very small concentration levels. In agreement with the Beer–Lambert law (Equation (1)), the ARHCF in fact acts as a 1 m long cuvette, which is integrated on the input side into a partially self-aligning monolithic optofluidic environment that can be accessed by state-of-the-art microfluidic infrastructure. Even though the RI of water is below that of silica, the presented fibers use the anti-resonant effect for light guiding, with the consequence of no post-processing of the fiber being required at any stage, yielding a waveguide system which is straightforward to use. The analyte volume required is three orders of magnitude lower than that needed for typical cuvette based systems, whereas the exchange of the entire liquid volume, including rinsing and refilling, is complete within seconds to minutes.

The optical characterization clearly showed that the introduction of water into the holey section of the ARHCF does not induce additional optical attenuation. Due to the different RI environment, the visible transmission band present in the unfilled case shifts towards the UV region in the case where the holes are filled with a water-based analyte. A key feature of our fiber is that UV guidance is provided in the transmission band between the resonances of orders *m* = 1 and *m* = 2, as this band yields the largest spectral bandwidth at a realistic strand thickness, which can be seen by inspecting Equation (2): The spectral bandwidth of a transmission band bordered by two adjacent strand resonances of the orders *m* and *m+1* is given by $\Delta \lambda =\lambda_{m}-\lambda_{m+1}=2\sqrt{n_{s}^{2}-n_{h}^{2}}t/\left(m+m^{2}\right)$. Considering that the resonance of order *m* should be located at the predefined resonance wavelength $\lambda_{R}$ (e.g., at UV wavelength), the spectral bandwidth equates to $\Delta \lambda =\lambda_{R}/\left(m+1\right)$.

The inverse dependence of $\lambda_{R}$ on m in this equation clearly suggests that high order transmission bands have smaller spectral extents; thus, it is favorable to operate in low order bands. Excluding the case that lowest order resonance (m=1) is located on the blue side of the measurement range ($\lambda_{R}<180\;\mathrm{nm}$), which would demand experimentally unfeasible thin strands (t<150 nm), the presented fiber represents the ideal compromise between using a low order resonance band (here m=1 and m=2) and experimentally realistic strand thicknesses (t>300 nm).

From the device and sensing perspectives, the integration of the ARHCF into an optofluidic environment yields a monolithic system that provides stable, reliable and efficient incoupling conditions, while simultaneously providing access to liquids, using a platform that is compatible with current microfluidics. Further steps in system integration will rely on including a second optofluidic chip on the other end of the ARHCF, which would yield more stable and mechanically more robust light outcoupling conditions. High quality linear calibration curves with R^2^ > 0.99 are obtained for both substances considered (sulfamethoxazole (SMX) and sodium salicylate (SS)), with the resulting absorbtivities matching those from simulations, showing that the absorption behaviour of substances located inside the ARHCF is well described by the Beer–Lambert law (Equation (1)). The sensing measurements showed limits of detection ($LoD_{SMX}=0.05\;µM$ and $LoD_{SS}=0.23\;µM$) that are comparable to those of other methods, including the performed double spectrometer measurements (Table 1). The sensing measurements of two application relevant test substances dissolved in water show detection limits (SMX: 0.1 µM, SS: 0.4 µM) that are comparable to those of other methods, including the performed double spectrometer measurements (Table 1). We attribute the comparable high detection limits to the discussed measurement-to-measurement variations imposed by the so far mechanically unfixed output butt coupling section, which has an impact on the determination of the analyte concentrations. As mentioned above, current experiments aim to improve measurement reproducibility by integrating the output section of the ARHCF into a second optofluidic port, yielding more stable and robust output conditions. For instance, assuming that the power fluctuations, in the case of SMX, reduce from currently 10%, to 1%, the LoD drops accordingly from 0.05 µM (12 µg/L) to 5 nM (1.2 µg/L), clearly outperforming the used Jasco spectrophotometer. It is important to note that other methods reach lower LoD values (ng/L regime) but are also more complex to apply, e.g., demand sophisticated sample preparation procedures (e.g., solid phase extraction (SPE) methods) that are labor-intensive and require well trained technical staff [4].

The concept of UV spectroscopy using optofluidically integrated ARHCFs yields a compact, straightforward, and transportable spectroscopic device that demands only very small volumes of liquid analyte, which can be exchanged in real-time. Since the current measurements suggest that the introduction of water into the holey sections of the ARHCF does not increase the loss, fiber lengths beyond the 1 m used in these measurements can be anticipated. Due to these unique properties, we believe that our concept will find application in various fields of liquid-based analytics, including environmental science and biomedicine.

## Figures and Tables

**Table 1:** Comparison of various detection methods for the quantitative identification of substance traces in aqueous solutions.

Method	Substance	LoD ^1^	Probe Volume	Source
UP ^2^ LC/MS/MS ^3^	SMX	0.5 ng/L	100 mL	Tamtam 2008 [32]
SPE ^4^ LC/MS/MS		0.1 ng/L	1 L	Loos 2007 [29]
SPE LC/MS/MS		7 ng/L	50 mL	Botitsi 2006 [33]
Abs. ^5^ spectroscopy in 1 cm cuvette		26 µg/L ^7^	2 mL	Jasco “V-700” spectrophotometer
Abs. spectroscopy in ARHCF		12 µg/L	10 µL	This paper
UV abs. spectroscopy	Aromatic compounds	64.900 µg/L	3.5 mL	Wittkamp 1997 [34]
SPME ^4^ + UV abs. spectroscopy		0.4.12 µg/L	50 mL	Wittkamp 1997 [34]
SPME + UV-EWA ^6^ spectroscopy		1..18 µg/L	10 mL	Merschman 1998 [35]

^1^ LoD = limit of detection. ^2^ UP = ultra performance. ^3^ LC/MS/MS = liquid chromatography electrospray tandem mass spectrometry. ^4^ SP(M)E = solid phase (micro)extraction. ^5^ Abs. = absorption. ^6^ EWA = evanescent wave absorption. ^7^ calculated from the spectroscopic accuracy value given by the instrument’s datasheet.

## Supplementary Materials

The following are available online at http://www.mdpi.com/1424-8220/18/2/478/s1, Figure S1: Xenon light source spectra and delivery loss spectrum.
