# Supplementary material for: UV Absorption Spectroscopy in Water-Filled Antiresonant Hollow Core Fibers for Pharmaceutical Detection
Source: Sensors (Basel). 2018 Feb 6;18(2):478. doi: 10.3390/s18020478 (PMC5855990; doi:10.3390/s18020478)
Supplement: Supplementary file 1 [file sensors-18-00478-s001.pdf]

Supplementary Material

Characteristics of light source and delivery fiber

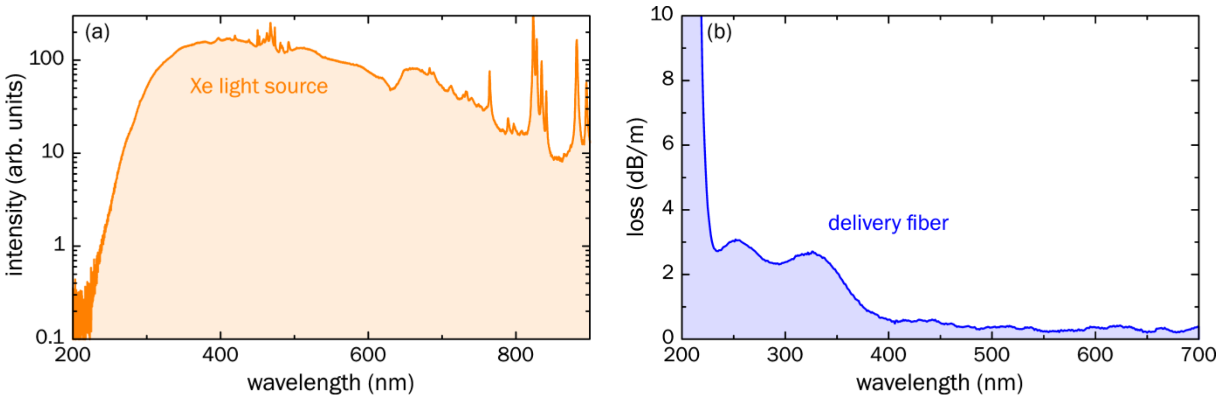

Fig. S1: *(a) Xenon light source spectra used for the UV spectroscopy experiments (incl. the contribution of around 35 cm length of UV-15/125 fiber). (b) Loss spectrum of the UV guiding delivery and collection fiber.*

Figure S1(b) presents the loss spectrum of the delivery fiber that was used for the light transport from source to sample, as well as from sample to spectrometer. It is a UV-transmitting solid-core fiber with 15 µm core and 125 µm outer diameter, a numerical aperture of 0.1 and tolerably low losses down to 230 nm, that was produced at the institute of the authors. The low numerical aperture and relatively small core size turned out to be advantageous for efficient light coupling to the ARHCF.

Derivation of the equation for the resonance wavelength (Eq.2)

The derivation of this equation relies on determining the spectral locations at which the guided modes inside the silica strand surrounding the central low index core go into cut-off, opening up loss channels for central modes to radially loose energy and thus to the emergence of strong loss peaks. To find these wavelengths, we assume that the silica strand surrounding the low index core section acts as a silica waveguide (refractive index n_s, thickness t) symmetrically embedded in a low index medium of index n_h. The different components of the wave vector of the modes inside that waveguide are interrelated by the following expression $(n_2 k_0)^2 = \beta_s^2 + k_{\perp,s}^2$ with the vacuum wave number k_0, the longitudinal invariant propagation β_s of the mode inside the medium s and the transverse wave number $k_{\perp,s}$. Guided modes impose that the transverse wave number is quantized as follows $k_{\perp,s} = \pi m/t$ with m being an integer number. At the cut-off (characterized by $\beta_s = n_h k_0$), the modes supported by the silica strand change their character from guided to leaky. These leaky modes allow the modes in the central low index core to transversely dissipate their energy, leading to extremely strong loss peaks in spectral distribution of the model attenuation of the central low index core mode. The wavelength at which the loss peaks emerge are obtained by inserting the cut-off and wave number quantization conditions into the above relation for the wave vector components, yielding Eq. 2.
